# Remarks on Dr. Kinglake's Observations on the Obstetric Practice

**Published:** 1816-01

**Authors:** J. Atkinson

**Affiliations:** Member of the Royal College of Surgeons in London.


*For the London Medical and Physical Journal.*

### *Remarks on Dr. Kinglake's Observations on the Obstetric Practice*; by J. Atkinson, Esq. Member of the Royal College of Surgeons in London.

IN the following observations I shall endeavour to divest myself as much as possible of all selfish considerations, being wishful to support the sacred cause of truth and the interests of humanity. If, upon an impartial examination, the obstetric practice shall be found to be derogatory to the dignity of science, or inimical to the dictates of genuine philanthropy, I shall not hesitate to abandon it (however lucrative) to illiterate midwives, or to those mercenary practitioners who imagine that the acquisition of riches can compensate for the loss of that peace of mind which should attend any gross deviation from the laws of virtue and honor.

With the greatest deference to the high professional character of Dr. Kinglake, I shall now proceed to examine his observations, and to substantiate my own upon facts which have occurred in my own practice.

Dr. Kinglake observes, "To suppose an inadequacy in Nature to accomplish every requisite respecting the birth of the human species, is to imagine that chance, and not consummate wisdom, is concerned in this most important event."

Of the supreme excellence, œconomy, and beauty of Nature, he will be the most thoroughly convinced who examines her the closest; nevertheless it cannot be denied, that her operations would often be incomplete if left solely to the influence of the immediate causes that govern them. For this purpose the human intellect is often necessarily brought in aid, as in the cultivation of the ground, the removing weeds or excrescences which would otherwise prevent or retard fructification; and in destroying noxious animals which would annoy the senses, or interrupt the well-being of the more important orders of organized beings; also, in the prevention of diseases, no one will dispute the advantages of vaccination; and, I think, it is easy to prove, that in no instance is the judicious interference of art more necessary than in alleviating the distresses or averting the dangers incident to human parturition.

It is true, indeed, the inferior animals require little or no aid, for the following plain reasons:—

1st, The heads of most animals are not nearly so spacious in proportion as the human head, in which it is well known that the brain is particularly large.

2dly, In animals the pelvis is placed horizontally, and therefore may conveniently be made larger; whereas in the human subject it is nearly vertical, which position must require a narrower form, in order that it may support its contents till the end of utero-gestation, otherwise prolapsus uteri and frequent abortions would be the consequence.

3dly, Animals are in a state of nature.

For these reasons, it is evident that human parturition must frequently be much more difficult and dangerous than that of animals, and particularly if we take into consideration that the former often do not begin to bear children till after the period intended by nature.

Dr. Kinglake thinks it would be better to confine man-midwifery to absolute occasions for operative aid. "Then," says he, "the art would be directly efficient." Thus he admits there are difficult cases, (notwithstanding the efficiency of nature,) which call for the assistance of art; but then, so far as instrumental aid is required, he limits to the three following conditions:—1st, wrong presentation; 2d, hæmorrhages; 3d, convulsions. It is well known, however, to every experienced accoucheur, that many other cases may and do occur, which imperiously require instrumental aid. Suppression of urine requires the introduction of the catheter; too large a size of the child's head requires the forceps or perforator. About four years ago, a lady, aged 40, had been attended, during her first labour, by a midwife, who suffered her to remain undelivered (though she had the most violent pains) for at least twenty-four hours after the ear of the child might be felt, and for that space of time the head had not moved perceptibly nearer the os externum. When I saw her, the pains had nearly ceased; pulse extremely weak, and countenance ghastly. After being convinced that nature was totally inadequate to the task, the operation of embryotomy was performed by myself and another practitioner, as the only means of giving the patient the smallest chance: in this way she was soon delivered of a very large child. Had it been done before the powers of life were exhausted, there can be little doubt that her life would have been saved; but, unfortunately, it was too late—she expired the following day. This is the only fatal case I have witnessed after this operation, although I have performed it several times How does this case accord with the following assertion of the doctor:—"Bad cases in midwifery are announced by features not to be mistaken; and, even on those occasions, the urgency is not so great, but there will be time sufficient to procure such assistance as may be required." It is, however, certain, that practitioners have frequently to regret that they have been called in too late, even in the present state of practice; but what would be the consequence, if the art was confined entirely to females, as no one can doubt that, besides a practical acquaintance with natural labour, an intimate knowledge of the situation of the parts is absolutely necessary, in judging of the case, or in affording relief in preternatural cases.

I shall now endeavour to prove the necessity of an accoucheur's attendance in all cases of midwifery. A medical man's attendance is principally required, in order to watch and be immediately apprised of any event that may require his assistance, as hæmorrhage, suppression of urine, preternatural presentation, &c. as these are much more easily remedied at the beginning, than after a lapse of time. About the commencement of my practice, a woman at Halton was attended by a midwife: a violent hæmorrhage came on before the head of the child was expelled, which had been aggravated by placing the patient near a large fire, and giving her a quantity of spirituous liquors, a custom by no means unfrequent among country midwives. The flooding became so violent, that the midwife ran out of the house, and said she durst not stay, but desired my immediate attendance. I happened at that time to be in the village: when I saw her, the blood was flowing from her in a continued stream, and the floor of the room was almost covered. By immediately extinguishing the fire, admitting a stream of cool air, and applying applying styptics to the pubes and surrounding parts, the discharge nearly ceased, and she was soon after delivered, though she has never recovered her former strength. I am pretty certain that, if the discharge had continued many minutes longer, it would inevitably have proved fatal.

A hæmorrhage is frequently produced by the presentation of the placenta, which, in general, is instantly suppressed by rupturing the membranes, an instance of which I witnessed not long ago, when attending a patient in Meadow-lane, in consultation with Mr. William Hey. Now, should the practice be confined to ignorant women, how seldom would a medical man be procured in time to prevent the fatal consequences of such accidents as these.

Sometimes Nature may be sufficient to accomplish the delivery, and yet, from the size of the head, the process may be so slow, as to occasion the bladder and vagina to slough; an instance of which is recorded in the London Practice of Midwifery. "In a consultation which was held in a case of this kind, it was agreed that Nature certainly would be able to deliver the woman; she, therefore, was not interfered with. She did deliver herself, but lost her life by it: she died, and that at a time when an ear was to be felt. This was certainly a case that required the use of the forceps, which would have delivered her with safety." Page 161.

It is well known, likewise, that the perinæum requires supporting, otherwise a laceration will often be the consequence, which not unfrequently renders the patient miserable for life. This, by skilful management, may, I believe, in all cases, be prevented. Sometimes the perinæum is stretched to such a degree, that it becomes as thin as paper.

An accoucheur's attendance is further necessary, to prevent the interference of ignorant persons, which is fully demonstrated by the foregoing cases. In have seen hæmorrhage induced by using too much force in separating the placenta; fevers and inflammation, by the exhibition of wine and spirits. Children have frequently been lost, when the feet have presented, by the ignorance of the midwife, in not being able to extract the head before the child was strangled; which extraction is easily effected, by introducing a finger into the mouth, and bringing the occiput forward, so as to correspond with the anatomical structure of the pelvis. Dr. Haighton mentions a case where a child's head was severed from its body by the force which a midwife used in its extraction.

A midwife is seldom competent to judge in what cases art is necessary, or how far it is proper to trust to the efforts of nature. It is not uncommon to see a patient, who has been two two or three days in labour, when the arm is the presenting part, and a vain hope has been entertained that Nature could effect the delivery, until the urgency of the symptoms, and the unaltered state of the parts, have proved that her struggles were unavailing. An intelligent practitioner would ascertain, at an early period, the nature of the case, bring down the feet, and deliver, without exposing the patient to all this unnecessary pain and danger.

I might say a great deal on the absolute necessity of art in various preternatural cases; but, as this is not questioned by the doctor, it will be unnecessary.

That Nature would, in many cases, complete the delivery with safety, there can be no doubt; but, if the following circumstances frequently occur, either for want of assistance, or by the unskilfulness of the assistant, it will justify the constant or uniform employment of well-educated men.

1st. Laceration of the perineum.

2d. Exhaustion of the strength, by undue stimulants or frightful tales.

3d. Hæmorrhage produced by mismanagement, as by separating part of the placenta by improper force; or, when accidental, of the necessity of restraining it.

4th. Bursting of the bladder, for want of a timely introduction of the catheter.

5th. Irreparable injury, by delay, in various preternatural cases.

6th. The omission of bleeding, and suitable remedies, in convulsions, puerperal fever, &c.

7th. Danger to the child, by suffering the funis to remain tight about its neck; in improperly dividing the naval string, so that the ligature shall embrace part of the intestines, or not of sufficient tightness to prevent a fatal hæmorrhage; or by a tardy or injudicious extraction of its head, when that is the part which remains last;—to say nothing about soothing the patient's mind, proper regulation of her diet, alleviation of certain symptoms by medical aid, position during labour, and many other circumstances.

Now, if the above accidents do often occur, and dangers to the patient's health and life, even in natural cases, often present themselves, which might easily be obviated by skilful management, (for the truth of which I appeal to practitioners in general,) then I think I shall have demonstrated that the practice of midwifery by scientific men is neither inconsistent with the unmolested comfort of the patient, the justice of philosophy, or the frankness of undisguised honesty.

*Meadow-lane, Leeds;*
*Nov.* 18, 1815.

